# Design and development of the MacTSQ measure of satisfaction with treatment for macular conditions used within the IVAN trial

**DOI:** 10.1186/s41687-018-0031-z

**Published:** 2018-02-07

**Authors:** Jan Mitchell, Clare Bradley

**Affiliations:** Health Psychology Research Unit, Orchard Building, Royal Holloway, University of London, Egham, TW20 0EX England

## Abstract

**Background:**

The purpose of the study was to design a measure of patient satisfaction with treatment for macular disease, the MacTSQ, and to carry out psychometric evaluation of the measure. The measure was designed along the lines of the widely used Diabetes Treatment Satisfaction Questionnaire (DTSQ) and sister measures of treatment satisfaction for other conditions including diabetic retinopathy. Information was also gathered during in-depth interviews with 20 people who had experienced one of a range of treatments for macular degeneration. In a prospective study, the newly designed 16-item MacTSQ, was used in a multi-centre, randomised, double-blind clinical trial (the IVAN study) comparing two treatments for neovascular age-related macular degeneration and two treatment schedules: 1. continual monthly treatments (continuous arm), 2. initial 3 monthly treatments then monitoring and retreatment if necessary (discontinuous arm). The MacTSQ was administered after the first three treatments and at 12 and 24 months. Psychometric development was carried out using data from 137 patients. Sensitivity and validity of the MacTSQ were investigated using baseline and 12-month data.

**Results:**

Exploratory factor analysis yielded two subscales i) convenience, information and overall satisfaction (6 Items: Cronbach’s alpha = 0.740), and ii) safety, efficacy and discomfort (6 Items: alpha = 0.776). Twelve items also loaded on to a single scale (alpha = 0.815). Three items were removed from the scale but retained in the questionnaire for separate analysis where required. Greater satisfaction was reported at time 2 (12 months) than time 1 (after 3 monthly injections) on the safety, efficacy and discomfort subscale (W = 3000.500. *p* = 0.024, *n* = 108). Participants whose vision improved reported greater satisfaction than those who had no improvement e.g. U = 1599, *p* = 0.033. Those in the discontinuous arm reported greater satisfaction on subscale 1 than those in the continuous arm at time one (U = 1870, *p* = 0.04) and time 2 (U = 1132.5, *p* = 0.023). This finding suggested a better experience in the discontinuous arm.

**Conclusions:**

The MacTSQ will be valuable in investigating treatment satisfaction in clinical trials of new treatments or in a routine clinic situation and may highlight ways to improve patients’ experience of treatment.

## Background

Macular degeneration is a chronic, degenerative disease of the eye which affects the centre of the retina, the macula. It mainly affects people over the age of 50 years, when it is referred to as age-related macular degeneration (AMD). It has been estimated that one in 30 people over 52 years old and one third of people over 75 years old have some degree of AMD [1]. It is the leading cause of blindness in people over 60 in the Western world [2] and incidence will increase in an ageing population [3]. AMD leads to loss of central vision needed for tasks such as reading, driving, watching television and recognising faces. The ability to carry out many day-to-day tasks is impaired [4] and AMD may compromise the ability to live independently. The psychological impact of the condition is considerable and can be devastating [4–6].

There are two types of AMD. The more common atrophic (dry) AMD accounts for around 80% of cases. This type of AMD progresses slowly and may not cause serious visual impairment. Currently there is no treatment for atrophic AMD. Neovascular (wet) AMD progresses rapidly and is responsible for 90% of cases of severe visual impairment due to AMD. Although there is no cure for neovascular AMD, treatments are available that may halt the progress of the condition for an indeterminate period [7] and can lead to improved vision for some patients [8].

With new treatments for AMD available and others being developed and assessed in clinical trials, it is important that an instrument is available to monitor patients’ satisfaction with their treatment. Patient satisfaction has been shown to influence patients’ health and treatment-related behaviours [9], including adherence to treatment [10] and this in turn will influence treatment outcomes. Lower levels of satisfaction with provision of information before treatment in other fields of medicine have been associated with increased depression post-treatment [11]. It is not inevitable that a treatment which results in a successful clinical outcome will be perceived as satisfactory by patients, particularly if the treatment is painful, unpleasant, inconvenient or associated with undesirable risks or side effects [9, 12].

The design of the MacTSQ measure of satisfaction with treatment for macular degeneration was modelled on the RetTSQ (measure of satisfaction with treatment for diabetic retinopathy) [13] which in turn was based on the DTSQ for diabetes [12, 14] and sister measures for other conditions (e.g. RTSQ [15], ThyTSQ [16]) which provide a template and item bank useful as a starting point in producing -TSQs for other conditions. The item content and layout of the RetTSQ was designed with the use of semi-structured interviews with patients with diabetic retinopathy of varying degrees of severity following various treatments. Interviews were conducted by psychologists in four centres, one each in Scotland and Southern England and two in Germany [17]. Ophthalmologists in all centres reviewed the content of the measure and had no further items to suggest. The final version of the RetTSQ was validated in a cross-sectional study of 207 German patients with diabetic retinopathy. They completed the RetTSQ during a clinic visit prior to their consultation and any treatment received. The validated RetTSQ was used as a basis for the MacTSQ. The MacTSQ was refined with the help of 20 one-to-one in-depth interviews with people with AMD. The final draft was used in a clinical trial, the IVAN trial [18]. IVAN compared two treatments, both intra-ocular injections of anti-VEGF medication (ranibizumab or bevacizumab) for neovascular AMD, and two treatment schedules: i) treatment monthly for 2 years or ii) treatment monthly for 3 months followed by monthly monitoring for 2 years and retreatment only if needed. This was a double blind trial with neither patients nor clinicians aware which of the two drugs was administered. Data obtained from the first post-treatment completion of the MacTSQ at 14 weeks were used to carry out psychometric development and to prepare a scoring algorithm. The present paper describes the design work conducted to produce the MacTSQ and the psychometric development of the instrument using data from the IVAN trial.

## Method

### Participants

The 20 people who took part in in-depth interviews were members of the UK Macular Disease Society (MDS, since renamed the Macular Society). They were recruited by one researcher during visits to MDS local group meetings and others were invited to take part at a Macular Disease Society national conference in London. Participants were selected so that as many different forms of AMD treatment as possible had been experienced by the participants interviewed.

Data used for the psychometric development of the MacTSQ were obtained from 137 patients newly diagnosed with neovascular AMD and recruited to IVAN at 10 of the 23 clinical sites participating in the trial.

Informed consent was obtained from all individual participants included in the study.

### Procedure

MacTSQ design interviews took place in the Department of Psychology, Royal Holloway. Two psychologists attended each interview, one conducting the interview and one taking notes. Interviews were recorded, with the participants’ permission. In the first part of the interview participants were asked about their experience of developing AMD, of being diagnosed, the path to treatment and their experience of their treatment(s). They were then asked to complete the MacTSQ, ‘thinking aloud’ while doing so in order to convey their understanding of the items. Suggestions for changes or additions to the questionnaire were invited and noted. Suggested changes to the MacTSQ were evaluated with future patients throughout the course of the interview phase.

In the double-blind clinical trial, participants completed the MacTSQ by telephone interview 1 week after their third treatment with intraocular injections of one of two anti-VEGF drugs. A second administration of the MacTSQ took place after the twelfth treatment/assessment appointment.

### MacTSQ

The MacTSQ first draft consisted of 13 items addressing specific aspects of treatment and one final item in which participants could note any aspects of treatment satisfaction that were not covered by the questionnaire. The layout of the questionnaire was designed to enable completion by people with visual impairment. Ariel 16 bold font was used throughout and all text was justified to the left. Response options were presented vertically.

Items are scored on a 7-point Likert-type scale from 6 (very satisfied) to 0 (very dissatisfied). For some items there is a ‘not applicable’ option e.g. in item 2 (*side effects and after effects of treatment*) the first response option is ‘no side effects experienced’. This response is scored as 7 for the purpose of recording frequencies but is recoded to 6 (very satisfied) for other analyses.

Question 10 (*provision of information about treatment*) is preceded by four questions, each with ‘yes’ and ‘no’ response options, asking:

‘Were you given any written information designed to prepare you for your MD treatment e.g. information about

1. the procedure
2. possible side-effects
3. expected benefits

10a. If yes, was the information given to you long enough before your treatment to allow you to make best use of it?’

These questions were intended to elicit additional data about the adequacy of information provision which was often reported to be unsatisfactory during the design work.

In the questionnaire the abbreviation ‘MD’ (macular degeneration) is used rather than AMD. In the UK people tend not to use the term ‘age-related’ and abbreviate the name of the condition to MD, which can refer to the broader category of macular disease as well as the more specific macular degeneration. This abbreviation would allow the instrument to be used for non-age-related varieties of macular degeneration such as Best’s disease or Stargardt’s disease, should treatments become available.

### Statistics

Exploratory factor analysis (EFA) and internal consistency reliability analysis (Cronbach’s alpha) were used to investigate the scale structure of the MacTSQ and to guide removal of items. The data were skewed, therefore non-parametric tests were used to compare subgroups (Mann Whitney) and to investigate changes over time (Wilcoxon signed ranks test).

## Results

### Design of the MacTSQ

The average age of the 20 interview participants (13 women, 7 men) was 71.1 years (s.d. 9.1). Average duration of AMD was 4.40 years (s.d. 2.55). Three people had dry AMD, 15 had neovascular AMD and two had neovascular in one eye and dry in the other. Three people were registered severely visually impaired, seven were visually impaired and nine were not registered (no data for one person). A wide range of treatments had been experienced including laser (*N* = 3), photo-dynamic therapy (7), Lucentis (ranibizumab) injections (3), Avastin (bevacizumab) injections (3), unspecified intra-ocular injection (1), acupuncture (1) and vitamin supplements (2).

As a result of the interview process two items were removed (*difficulty of treatment* and *influence over treatment*) as they were not well understood and, as they originated from the RetTSQ, more relevant to diabetic retinopathy; three were added (*cost of treatment, experience of fluorescein angiogram* and *journey to the clinic)* and one item was expanded to become two items (*time consuming* replaced by *time at hospital on treatment day* and *time taken by course of treatment*). Minor changes were made to several items to improve comprehensibility. The 15 domains investigated in the final draft of the MacTSQ are shown in Table 1. A final item asked whether the respondent had any reasons for satisfaction or dissatisfaction that were not contained in the questionnaire. A space to describe was provided for those responding ‘yes’.

**Table 1:** Range of response options used at time 1 and mean scores for each item. Subscale and single scale means and medians at time 1 and time 2 and comparison of time 1 and time 2 scores

		Range of response options used	Time 1 mean (sd) *N* = 137	Time 1 median (range)	Time 2 mean (sd) *N* = 110	Time 2 median (range)
1^b^	Treatment for MD	3-6	5.61 (0.79)	6 (3-6)	5.62 (0.71)	6 (3-6)
2^ab^	Side effects and after affects of the treatment	0-7	5.28 (1.13)	6 (1-6)	5.39 (1.04)	6 (1-6)
3^ab^	Pain or discomfort associated with the treatment	0-7	5.17 (1.34)	5.5 (0-6)	5.24 (1.18)	6 (0-6)
4^bc^	How well the treatment is working	2-6	4.84 (1.24)	5 (2-6)	5.24 (1.21)	6 (0-6)
5^b^	How unpleasant treatment is	0-6	4.97 (1.29)	5 (0-6)	5.10 (1.24)	5.5 (0-6)
6^b^	Apprehension about treatment	0-6	5.05 (1.41)	6 (0-6)	5.19 (1.38)	6 (0-6)
7^a^	Cost associated with treatment	0-7	5.62 (1.05)	6 (2-6)	5.79 (0.69)	6 (2-6)
8	Journey to the eye clinic for treatment	0-6	5.13 (1.35)	6 (0-6)	5.13 (1.23)	6 (0-6)
9^b^	Safety of the treatment	3-6	5.40 (0.97)	6 (3-6)	5.58 (0.67)	6 (3-6)
10^b^	Provision of information about the treatment	2-6	5.65 (0.73)	6 (2-6)	5.61 (0.80)	6 (2-6)
11^b^	Continuing or repeating treatment	3-6	5.64 (0.74)	6 (3-6)	5.46 (1.11)	6 (0-6)
12	Experience of fluorescein angiogram	2-6	5.35 (0.87)	6 (2-6)	5.45 (0.99)	6 (0-6)
13	Time at hospital on treatment day	0-6	4.33 (1.44)	4 (0-6)	4.35 (1.39)	5 (0-6)
14	Time taken by course of treatment	2-6	4.97 (1.12)	5 (2-6)	5.22 (1.24)	6 (0-6)
15^b^	Encourage others to have treatment	3-6	5.77 (0.70)	6 (3-6)	5.77 (0.63)	6 (3-6)
16^b^	Other aspects of treatment not covered by the questionnaire	n/a	n/a		n/a	
Subscale 1 scores (*N* = 108)			32.34 (3.56)	33 (17-36)	32.09 (4.11)	33 (17-38)^d^
Subscale 2 scores (*N* = 108)			30.86 (5.04)	32 (10-36)	31.75 (5.16)	34 (10-38)^e^
Single scale scores (*N* = 110)			63.20 (7.30)	65 (38-72)	63.84 (8.30)	67 (27-73)^d^

Items marked

^a^have 8 response options, all others have 7. Recoded data were used to calculate means and median for these items

^b^Items adopted from RetTSQ

^c^Item scores improved significantly between time 1 and time 2

^d^Comparison of time 1 and time 2 scores n/s

^e^Comparison of time 1 and time 2 scores: Z = 2.253, *p* = 0.012

### Clinical trial data

A total of 137 participants completed the MacTSQ about 1 week following their third treatment in the clinical trial (see Table 2 for participant characteristics). Follow-up MacTSQ data were collected at 12 months, by which time 110 of the 137 participants remained in the trial (deceased = 5, exited study prior to second MacTSQ completion = 6, unable to complete within the required time for second completion of MacTSQ = 6, unknown reason for leaving study = 10).

**Table 2:** Participant characteristics

	Men	Women
N	48	89
Mean age (sd)	76.9 (6.04)	77.8 (7.08)
VAR in study eye > fellow eye	9	22
VAR in fellow eye > 70 letters^a^	33	56
Continuous treatment arm	26	43
Discontinuous treatment arm	22	43

*VAR* visual acuity rating

^a^Snellen 20/80

### Psychometric development of the MacTSQ

Descriptive data obtained from the MacTSQ baseline dataset of the IVAN trial indicated that the full range of response options was used for seven of the 15 items (Table 1). For four items the scores ranged between 2 and 6 and between 3 and 6 for the remaining four items. Mean scores for individual items at time 1 ranged between 4.33 (*time spent at hospital on treatment day*) and 5.77 (*encourage others to have the treatment*), indicating high levels of satisfaction overall (see Table 1 for time 1 and time 2 means).

### Factor structure and reliability

Prior to conducting the main exploratory factor analyses (EFA) an unforced principal components analysis (PCA) was run. Output from the PCA was used to check the data to ensure suitability for factor analysis. The unforced PCA included 15 items and responses from 134 participants (listwise deletion excluded three participants). Inspection of the correlation matrix revealed no inter-item correlation exceeding *r* = 0.8, thus indicating no problems with multicollinearity within the data. Item 12 ‘*experience of fluorescein angiogram*’ was noted as potentially problematic with all correlations < 0.2. Inspection of the Measure of Sampling Adequacy (MSA) coefficients however revealed a value of 0.660 and therefore above the cut-off point of 0.60 [19]. It was decided not to remove Item 12 at this point. All other MSA values were 0.683 or greater. The Kaiser-Meyer-Olkin (KMO, measure of sampling adequacy) value was 0.777, comfortably exceeding the cut-off value of 0.60 [20, 21]. Bartlett’s Test of Sphericity [22] reached statistical significance. All data checks indicated that the properties of the correlation matrix justified a factor analysis being carried out.

On completion of all data checks the component loadings were examined. Factors were considered for retention using three decision rules: 1) Kaiser’s criterion (eigenvalues > 1), 2) inspection of the scree plot and 3) Horn’s parallel analysis [23]. The PCA revealed four components with eigenvalues greater than 1. Examination of Cattell’s scree plot suggested a possible three component solution and parallel analysis [23, 24] also suggested a three component solution. The decision was taken to explore a three factor solution for the EFA based on the consistent findings from the scree plot and the Parallel Analysis and that the eigenvalue > 1 rule has been shown to be the least accurate for assessing factor retention [25, 26].

Once the number of factors for extraction had been decided, the factor structure was explored using a forced three-factor Principal Axis Factoring (PAF) with oblique rotation (to allow for potential correlation between the factors). PAF was chosen as an extraction method due to the non-normal distribution of the data [27].

The initial EFA, run using all 15 items on the MacTSQ, revealed a clean three-factor structure. However, Item 12 ‘*fluorescein*’ was found to be a low loading item, loading highest on factor 3 at 0.141. Examination of the factor correlation matrix revealed factor 1 and factor 2 correlated at − 0.439 and factor 1 and factor 3 correlated at 0.322 therefore justifying the use of an oblique rotation. Item 12 ‘*flourescein*’ was removed and a forced three PAF analysis was run with the remaining 14 items. The analysis again revealed a clean three-factor structure, however factor 3 contained only two items. As a factor with fewer than three items is more likely to be weak and unstable [25], a forced two-factor solution was run with the 14 items. The two items previously on factor 3 (Item 7 ‘*cost*’ and Item 8 ‘*journey*’) now loaded on factor 1. Item 7 ‘*cost*’ however loaded < 0.3. Item 7 was removed and a forced two PAF run including the remaining 13 items. The analysis revealed a clean two-factor structure however Item 8 ‘*journey*’ and Item 4 ‘*how well is the treatment working*’ loaded < 0.4. Item 8 loaded less strongly and was removed. A forced two-factor PAF was run with 12 items. This final run revealed a clean two-factor structure comprising 12 items, all items load > 0.4 except for Item 4 ‘*how well is the treatment working*’ which loaded at 0.333. Although loading less than 0.4 the decision was taken to retain this item due to its importance to the content of the scale. The variance explained for the two-factor solution was 38%. The two factors were 1) Information and convenience and overall satisfaction and 2) Safety, efficacy and discomfort (see Table 3).

**Table 3:** Pattern and structure matrix for principal axis factoring with oblimin rotation of two-factor Solution of MacTSQ items

		Pattern coefficients		Structure coefficients	
Item N	Item	1	2	1	2
11	Continue	*0.728*	0.002	*0.728*	−0.340
10	Information	*0.642*	0.145	*0.621*	−0.286
15	Encourage others	*0.623*	0.006	*0.619*	−0.399
14	Length of course	*0.553*	−0.140	*0.573*	−0.155
1	Treatment	*0.513*	−0.097	*0.559*	−0.337
13	Time at clinic	*0.468*	−0.066	*0.498*	−0.285
6	Apprehensive	0.059	*−0.716*	0.395	*−0.744*
3	Discomfort or pain	−0.018	*−0.678*	0.300	*−0.670*
9	Safety	−0.086	*−0.655*	0.513	*−0.646*
2	Side effects	−0.008	*−0.541*	0.221	*−0.615*
5	Unpleasant	0.270	*−0.519*	0.245	*−0.537*
4	Treatment working	0.252	*−0.333*	0.408	*−0.451*

Variance = 38.62

Italicized figures represent the pattern and structure coefficients of the 2-factor solution

In order to explore internal consistency reliability Cronbach’s alpha analyses were run. As shown in Table 4, subscale 1 and 2 demonstrate good reliability, with both six-item subscales achieving an alpha value exceeding 0.7.

**Table 4:** Internal consistency reliability for each of the two MacTSQ subscales

Item N	Item	Cronbach’s alpha if item deleted	
		Information provision and convenience	Safety, efficacy and discomfort
11	Continue	0.683	
10	Information	0.706	
15	Encourage others	0.668	
14	Length of course	0.713	
1	Treatment	0.716	
13	Time at clinic	0.734	
6	Apprehensive		0.709
3	Discomfort or pain		0.728
9	Safety		0.735
2	Side effects		0.745
5	Unpleasant		0.759
4	Treatment working		0.774
Subscale alpha		*0.740*	*0.776*
Eigenvalues		*4.477*	*1.652*

Italicized figures represent the pattern and structure coefficients of the 2-factor solution

In order to provide the broadest possible single indicator of treatment satisfaction a forced one-factor exploratory factor analysis (EFA) was run. The forced one-factor PAF included 12 items. All items loaded > 0.4 and explained 29.25% of the variance in the data. Cronbach’s alpha test of internal consistency reliability was strong with an alpha of 0.815 for the 12-item scale (see Table 5).

**Table 5:** MacTSQ Single scale forced one factor: Principal axis factoring factor loadings and internal consistency

Item N	Item	Factor loadings	Cronbach’s alpha if item deleted
9	Safety	0.680	0.790
6	Apprehensive	0.637	0.788
11	Continue	0.612	0.800
14	Length of course	0.600	0.794
3	Discomfort or pain	0.540	0.796
1	Treatment	0.531	0.804
15	Encourage others	0.528	0.806
4	Treatment working	0.508	0.803
13	Time at clinic	0.464	0.810
2	Side effects	0.461	0.804
5	Unpleasant	0.442	0.806
10	Information	0.420	0.811

Variance = 29.26

Cronbach’s Alpha = 0.815

### MacTSQ scoring and mean scores

Each subscale has 6 items scored between 6 (e.g. very satisfied) and 0 (e.g. very dissatisfied). Item scores are summed to give a subscale score of between 0 and 36. The single scale has 12 items, each scored between 6 and 0. Items are summed to give a score of between 0 and 72. For all three scales, the higher the score, the greater the satisfaction with treatment.

### Missing data

Analyses were carried out to establish how many items of missing data could be present before the alpha fell below 0.7. For each subscale and the single scale, the item causing greatest detriment to alpha if removed was removed from the scale and the reliability analysis re-run. This was repeated until the removal of another item would result in the alpha falling below 0.7. Using this procedure, it was established that for subscale 1, no missing data could be tolerated, for subscale 2, one item of missing data could be tolerated and, for the single scale, up to three items of missing data could be tolerated before alpha fell below 0.7. Cases with more than the maximum acceptable number of items of missing data should be removed from the dataset before analysis. Where there are missing data for one item only on subscale 2 and/or up to three items missing on the total scale the missing scores can be replaced with means for the remaining items on that subscale/total scale for the purposes of computing the subscale score or total score. Missing scores are best treated as missing when examining means and medians of single item scores.

### Sensitivity of the MacTSQ

Subscale scores at Times 1 and 2 were compared for the entire sample (Table 1). Subscale 1 and the single scale showed no change between times 1 and 2. Subscale 2 showed higher satisfaction at time 2 (time 1 median = 32, time 2 median = 34; Z = 3000.500. *p* = 0.024, *n* = 108). Comparison of continuous and discontinuous arms of the study showed that the discontinuous arm reported higher scores in subscale 1 at times 1 and 2. No such differences between treatment arms were noted for subscale 2. Single scale scores were higher for the discontinuous arm at time 2 (Table 6).

**Table 6:** Comparison of subscale and single-scale scores between continuous and discontinuous arms at times 1 and 2

	Median scores		
Treatment arm	Continuous	Discontinuous	statistic
Time 1 subscale 1	32	33.5	U = 1870, *p* = 0.04
Time 2 subscale 1	33	34	U = 1132.5, *p* = 0.023
Time 1 subscale 2	32	33	N/S
Time 2 subscale 2	34	34	N/S
Time 1 single scale	64	66	N/S
Time 2 single scale	65	68	U = 1126.5, *p* = 0.021

Subscale and single-scale scores were compared between groups with different visual outcomes at time 2. Three comparisons were made: any letters gained vs no letters gained or letters lost; 15 or more letters gained vs fewer than 15 letters gained; 15 or more letters gained vs letters lost. Subscale 1 showed no differences between the groups but subscale 2 distinguished between the groups in all three comparisons, showing greater satisfaction for those with improved vision. The single scale distinguished similarly between the groups in two out of the three comparisons (Table 7).

**Table 7:** Comparison of MacTSQ scores amongst subgroups with gain or loss of letters at 12 months

	Subscale 1		Subscale 2		Single scale	
	medians	statistic	medians	statistic	medians	statistic
Gained letters Loss or no gain of letters	34 33	N/S	34 33	U = 1788 *p* = 0.007	68 63	U = 1599 *p* = 0.033
Gained ≥ 15 letters Gained < 15 letters	34.5 33	N/S	35 34	U = 1220.5 *p* = 0.033	68.5 66	N/S
Gained ≥ 15 letters Lost letters	34 33	N/S	35 33	U = 588 *p* = 0.005	63 68.5	U = 553 *p* = 0.039

Comparison of single-item scores at time 1 and time 2 showed only two significant differences:

First, *How well do you feel your treatment is working: n* = 105 (T1 median = 5, mean = 4.85; T2 median = 6, mean = 5.22) Z = 1185.500, *p* = 0.009. The result suggests that some participants had a gradual improvement in vision.

Secondly, *If further treatment for your MD were necessary, how satisfied would you be to continue or repeat the treatment: n* = 108, (T1 median = 5, mean = 5.7; T2 median = 5, mean = 5.5), Z = 161.500, *p* = 0.049. This second finding indicates that some may have found the year-long course of treatment wearing. This may particularly have been the case for those who felt they were not benefitting from the treatment.

In response to the questions relating to provision of information (Item 10), six people reported that they had not been given written information about procedures, 13 said they were not told of possible side-effects and 27 participants indicated that they were not told of expected benefits. Information about both risks and benefits was given in the patient information letter and these data suggest either that some participants did not read their letter or have it read to them or that, by the time they completed the questionnaire, they had forgotten the details of the letter.

## Discussion and conclusions

The MacTSQ measure of macular disease treatment satisfaction was based on the RetTSQ measure of treatment satisfaction in diabetic retinopathy [17] and was adapted with the help of 20 people who had been treated for MD with a range of interventions. The contributions of people with MD ensured the content validity of the measure and their assistance during in-depth interviews enabled the developers of the measure to establish its face validity.

EFA resulted in the removal of three items, all of which had been added during the interview process (rather than existing items from the item bank). Without these three new items, the two subscales were obtained with a forced 2-factor analysis which yielded satisfactory factor loadings. A forced 1-factor solution also yielded satisfactory loadings. The first subscale, encompassing *convenience, information and overall satisfaction*, includes items relating to the clinic management as well as overall satisfaction with treatment and may be useful in informing improvements to clinic services. The second subscale, *safety, efficacy and discomfort,* contains items which investigate important aspects of the patient’s personal experience of the actual treatment and perceptions of its safety and it will provide useful feedback for clinicians and pharmaceutical companies about the acceptability of treatment for the patient. The single scale will give an overall picture of the patient’s view of the treatment being investigated.

Three items were eliminated from the scale but retained in the questionnaire to be analysed separately if required. *Cost of treatment* was retained because, whereas in the clinical trial all treatment was free, in other circumstances and in some other countries, the treatment being assessed may not be freely available. In the present study some people reported being dissatisfied with the cost of treatment. It is likely that those people had paid privately for treatment in the fellow eye prior to the clinical trial. *Satisfaction with fluorescein angiogram* appeared to have led to confusion for a small number of participants (*N* = 5) who reported that they had not had the diagnostic test, even though all participants in the trial did have the test. This item would be included in the MacTSQ and analysed separately if clinics particularly wanted to investigate satisfaction with fluorescein angiogram. *Journey to the eye clinic* was not included in the MacTSQ scales because it did not load on any scale or subscales satisfactorily and the item content is not directly relevant to satisfaction with a particular treatment. However, five people reported being very dissatisfied with their journey to the eye clinic and a total of 11 people scored between 0 and 2. Some people may have to travel long distances to reach hospitals where a treatment is available and, for elderly people who do not have ready access to transport, this can present considerable difficulties. This again is an item that could be included in the questionnaire and analysed separately if the issue of accessibility of treatment was under investigation.

The Cronbach’s alpha measure of internal consistency reliability yielded satisfactory scores for each of the subscales and the entire scale.

The full range of response options was used for seven items, the three most extreme levels of dissatisfaction (scores of 0 – 2) were not used in four items and the two most extreme indications of dissatisfaction (scores of 0-1) were not used in four items. Overall the data were negatively skewed. Atkinson et al. (9) commented that reports of skewness and ceiling effects are particularly common in the patient satisfaction literature. This might raise concerns that the MacTSQ would be insensitive to improvements in satisfaction but, in spite of the skewed data, investigations into the sensitivity of the measure were promising.

The satisfaction score for subscale 2 (*safety, efficacy and discomfort*) improved between time 1 and time 2. This may be expected as patients become more familiar with the treatment and less concerned about possible risks. The single scale showed that there was no difference in satisfaction between the continuous and discontinous treatment groups at time 1 but showed greater satisfaction reported by the discontinuous treatment group at time 2. Some of the discontinuous treatment group would not be having injections every month and so would avoid any negative psychological or physical impact of the treatment when injections were not necessary. Also, if examination revealed that treatment was not required, this would result in less time being spent at the hospital on that day than if treatment was carried out. This is likely to be reflected in higher satisfaction scores. The results offer encouraging early evidence for the sensitivity of the MacTSQ. Further evidence for the sensitivity of the MacTSQ to differences in satisfaction with treatment is demonstrated by satisfaction ratings associated with changes in vision during the trial.

The questions contained in item 10 (*provision of information)* give additional information about the provision of information apart from the respondent’s estimate of his/her satisfaction. The data from this study indicate that some participants did not recall being given information about the procedure or the potential risks, side-effects and benefits associated with it. All relevant information would, most probably, have been provided in the patient information letter. In the context of the clinical trial it is highly unlikely that participants were not given the information in the form of a patient information letter and the findings highlight the necessity of checking that patients have read and understood the information provided and, if not, providing an oral account. When the MacTSQ is used in other situations, where less stringent routines are adopted, it will be useful in drawing attention to any shortcomings in information provision. Even in a clinical trial it is possible that the protocol may not be followed for every patient in every centre.

The open question at the end of the MacTSQ elicited comments from 53 participants at the time 1 data collection. No new areas of satisfaction/dissatisfaction arose. Many comments were complimentary towards doctors and nursing staff. The most common criticisms concerned waiting times and discomfort experienced during or after the treatment, both issues already covered by existing MacTSQ items.

Although the initial draft of the MacTSQ was not designed using in-depth interviews to develop an initial bank of items, it was based on a similar measure for diabetic retinopathy (the RetTSQ) which was developed using that method. The early draft of the MacTSQ was refined using in-depth interviews and the ‘think aloud’ technique and so the design was directly influenced by the contributions of people who had been treated for MD. The fact that no new areas of satisfaction or dissatisfaction were elicited from the open question attests to the content validity of the measure.

In the IVAN trial, the MacTSQ was administered by telephone interview. Although there is evidence of potential differences in scores according to the method of administration [28] there would have been no discrepancy within the trial as the same method was used throughout. Telephone interviews have been used successfully in other studies of this population e.g. [28] and it is likely to result in a better response rate and shorter completion time since some participants’ vision may have made completing a paper version difficult. No information about completion time was given in the IVAN trial report [18] but experience suggests that telephone completion of the 13-item MacTSQ is unlikely to exceed 12 min.

An important quality in any PRO is test-retest reliability, which demonstrates the extent to which participants’ scores are stable over a period during which no change in their condition occurs. In the IVAN trial the MacTSQ was used at 3 months and 12 months and this long interval between applications, and the likelihood of changes in vision, precluded the investigation of test-retest reliability. It will be valuable to investigate test-retest reliability in future research.

It would have been helpful to have collected MacTSQ data after the first treatment but this was not in the clinical trial protocol. It would be expected that participants would experience considerable apprehension before their first experience of an intra-ocular injection but that they would report less apprehension subsequently if the first injection was not unpleasant. For the purposes of the present work, no access was given to data concerning participants’ randomisation to either ranibizumab or bevacizumab.

The IVAN trial studied two similar treatments for one macular condition, neovascular AMD. Further validation work will be necessary to establish the MacTSQ’s suitability as a measure of treatment satisfaction for other macular conditions and other treatments.

Published 2-year results from the IVAN study reported no difference in MacTSQ scores between the two subgroups, though only the single scale was analysed and only at the 2-year time point [18]. The data reported here indicate that investigation of subscale scores at different time points can give a more fine-grained picture of patients’ experience of and satisfaction with treatment. Since this study, other research using the MacTSQ has shown it to be a useful tool in assessing patients’ satisfaction with treatment for myopic choroidal neovascularization [29]. The MacTSQ is a short measure which investigates a variety of aspects of treatment satisfaction. It is designed to be suitable for all macular conditions in adults including neovascular age-related macular degeneration. It is suitable for use as a single scale and/or as two subscales and scrutiny of individual items will provide useful information. The MacTSQ will be valuable in investigating treatment satisfaction in clinical trials of new treatments or in a routine clinic situation and may highlight ways in which treatment satisfaction can be improved.
